# Enabling structure-based drug design of Tyk2 through co-crystallization with a stabilizing aminoindazole inhibitor

**DOI:** 10.1186/1472-6807-12-22

**Published:** 2012-09-20

**Authors:** Maria A Argiriadi, Eric R Goedken, David Banach, David W Borhani, Andrew Burchat, Richard W Dixon, Doug Marcotte, Gary Overmeyer, Valerie Pivorunas, Ramkrishna Sadhukhan, Silvino Sousa, Nigel St John Moore, Medha Tomlinson, Jeffrey Voss, Lu Wang, Neil Wishart, Kevin Woller, Robert V Talanian

**Affiliations:** 1 Department of Molecular & Cellular Pharmacology, Abbott Laboratories, Worcester, MA, USA; 2 Present Address: D. E. Shaw Research, New York, NY, USA; 3 Department of Chemistry, Abbott Laboratories, Worcester, MA, USA; 4 Present Address: Vertex Pharmaceuticals, Cambridge, MA, USA; 5 Present Address: Department of Physical Biochemistry, Biogen Idec, Cambridge, MA, USA; 6 Department of Biologics, Abbott Laboratories, Worcester, MA, USA; 7 Department of Molecular Cell Biology, Harvard University, Cambridge, MA, USA; 8 Advanced Technologies, Abbott Laboratories, Abbott Park, IL, USA

**Keywords:** Tyk2, Jak kinase, Crystallization, Proteolysis

## Abstract

**Background:**

Structure-based drug design (SBDD) can accelerate inhibitor lead design and optimization, and efficient methods including protein purification, characterization, crystallization, and high-resolution diffraction are all needed for rapid, iterative structure determination. Janus kinases are important targets that are amenable to structure-based drug design. Here we present the first mouse Tyk2 crystal structures, which are complexed to 3-aminoindazole compounds.

**Results:**

A comprehensive construct design effort included *N*- and *C*-terminal variations, kinase-inactive mutations, and multiple species orthologs. High-throughput cloning and expression methods were coupled with an abbreviated purification protocol to optimize protein solubility and stability. In total, 50 Tyk2 constructs were generated. Many displayed poor expression, inadequate solubility, or incomplete affinity tag processing. One kinase-inactive murine Tyk2 construct, complexed with an ATP-competitive 3-aminoindazole inhibitor, provided crystals that diffracted to 2.5–2.6 Å resolution. This structure revealed initial “hot-spot” regions for SBDD, and provided a robust platform for ligand soaking experiments. Compared to previously reported human Tyk2 inhibitor crystal structures (Chrencik *et al.* (2010) *J Mol Biol* 400:413), our structures revealed a key difference in the glycine-rich loop conformation that is induced by the inhibitor. Ligand binding also conferred resistance to proteolytic degradation by thermolysin. As crystals could not be obtained with the unliganded enzyme, this enhanced stability is likely important for successful crystallization and inhibitor soaking methods.

**Conclusions:**

Practical criteria for construct performance and prioritization, the optimization of purification protocols to enhance protein yields and stability, and use of high-throughput construct exploration enable structure determination methods early in the drug discovery process. Additionally, specific ligands stabilize Tyk2 protein and may thereby enable crystallization.

## Background

Janus kinases (Jaks) have broad roles in immune regulation via their action in cytokine signalling [1-3]. These non-receptor tyrosine kinases phosphorylate receptor chains, which in turn recruit and phosphorylate members of the Signal Transducer and Activator of Transcription (STAT) family [2,4]. The Jak family comprises Jak1, Jak2, Jak3 and Tyk2. These enzymes have very similar domain structures, containing a FERM domain, an SH2 domain, a pseudokinase domain, and a catalytic tyrosine kinase domain. Jaks serve overlapping but distinct functions in cytokine signaling, as demonstrated by knockout, mutation and other studies [5-9].

Because of their roles in the signaling of many important cytokines, hormones, and growth factors such as IL-2, IL-4, IL-6, IL-7, IL-12, IL-13, IFN-α, IFN-γ, Epo, and GM-CSF [10,11], Jak inhibitors might have wide application in the treatment of inflammatory, myeloproliferative and autoimmune diseases, and therefore the Jak enzymes are attractive targets for drug discovery. Initial studies with Jak3 inhibitors were aimed at preventing solid organ transplant rejection [12,13]. More recent studies have explored the potential of such compounds in chronic autoimmune diseases such as rheumatoid arthritis and psoriasis [14-16]. For example, tofacitinib (CP-690,550), which inhibits Jak1, Jak2, and Jak3, has demonstrated efficacy in Phase II trials for rheumatoid arthritis [17-19]. Ruxolitinib (Jakafi®), a dual Jak1 and Jak2 inhibitor [20], was recently approved for the treatment of myelofibrosis, a disorder involving myeloproliferative neoplasm.

The development of Tyk2 inhibitors is less advanced. Tyk2 functions together with Jak2 in the signaling of IL-12 and IL-23 via its interaction with the IL-12Rβ1 receptor chain, and in the coordinated phosphorylation of STAT3 & STAT4 [4,21]. Human Tyk2 gene deficiency causes defects in signaling of multiple cytokines, including IL-6, IL-10, IL-12 and IL-23, and reduced production of IFNγ [5]. Furthermore, Tyk2-deficient mice are resistant to experimental autoimmune encephalomyelitis, a model for multiple sclerosis [22,23]. Given the importance of Tyk2-dependent downstream cytokine signaling in this and other diseases such as rheumatoid arthritis and Crohn’s disease, Tyk2 inhibitors have the potential to be important therapeutics.

Because Jak family active sites exhibit high sequence identity, designing inhibitors selective within the family is challenging. One way to approach this challenge is to target active site regions that differ in conformation between homologs. To identify these “hot-spot” regions, we set out to obtain multiple crystal structures of Tyk2 in complex with a variety of ligands representing diverse chemotypes. At the time of our initial work, only Jak2 and Jak3 crystal structures had been published [24,25]. Robust Tyk2 crystallography allowing for the soaking of multiple inhibitors, essential for rapid throughput in structure-based drug design, had not been described. After exploring multiple constructs, we obtained crystals of mouse Tyk2 in the presence of 3-aminoindazole inhibitors that diffracted to 2.5–2.6 Å resolution. The inclusion of a ligand was absolutely required to obtain high-quality crystals, and we found through limited proteolysis experiments that the enzyme is significantly stabilized by binding to such ATP-competitive inhibitors. This process enabled the determination of multiple inhibitor-soaked Tyk2 crystal structures, forming the basis of an extensive SBDD program.

## Results and discussion

### Construct design and purification strategies

Several strategies were employed to obtain sufficient protein purification yields for crystallization: (1) variation of *N*- and *C*-terminal boundaries of the Tyk2 catalytic domain (some constructs included the pseudokinase domain); (2) variation of the affinity purification tag; (3) introduction of a kinase-inactivating mutation; and (4) use of multiple orthologs. Table 1 lists the different strategies and examples employed for Tyk2 construct design. After exploring roughly 40 constructs (list shown in Additional file 1 Table 1), we prioritized a mouse construct that produced adequate amounts of soluble protein for crystallization (GST-Tev-muTyk2 (870–1170) Asp1016Ala). The human and mouse Tyk2 catalytic domain sequences are highly conserved (91% identity in catalytic domain, 78% identity overall); however, several divergent surface residues had the potential to impact protein aggregation and crystallization behavior. A glutathione-S-transferase (GST) tag was included to increase solubility during early stages of purification, and the Asp1016Ala kinase-inactive mutation was introduced to increase conformational homogeneity by preventing multiple phosphorylation states; this mutation also increased expression approximately three-fold (data not shown). Asp1016 is the conserved catalytic base that is essential for phosphotransferase activity in protein kinases [26].

**Table 1:** Representative Tyk2 expression constructs

**Rationale**	**Constructs**
Orthologs	Human (hu)
	Mouse (mu)
Variation of affinity tag	His-Tev-huTyk2 (891–1185)
	His-Tev-huTyk2 (880–1185)-Flag
	GST-Thrombin-huTyk2 (870–1180)
	GST-Tev-muTyk2 (870-1170)
*N*- and *C*-terminal variations	huTyk2 (880–1185) huTyk2 (891–1185)
	huTyk2 (891–1175)
	huTyk2 (589–1185)
Kinase-inactive mutations	huTyk2 (870–1180) D1023A
	muTyk2 (870-1170) D1016A

**Figure 1:**
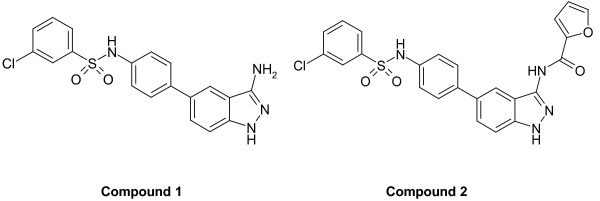
Structure of 3-aminoindazole inhibitors.

Previous attempts to purify the human Tyk2 protein using multiple chromatographic steps resulted in low yields or no detectable protein. Due to the aggregation and solubility problems seen with the human isoform, orthologs were considered and an abbreviated purification protocol was implemented. This protocol entailed batch binding to GST resin for several hours, followed by a resin wash and an “on column” TEV protease cleavage step. A critical step was to introduce the ligand (Compound 1) (Figure 1) at low protein concentrations, to prevent precipitation, and subsequently to co-concentrate the Tyk2/Compound 1 complex to a level useful for crystallization trials. Compound 1 (IC_50_ 6 nM against human Tyk2; Table 2) was one of the few inhibitors that co-crystallized with mouse Tyk2, allowing us to determine the structure of the mouse Tyk2 kinase domain. We also present the structure of Compound 2 complexed to mouse Tyk2, which was solved using inhibitor soaking methods.

**Table 2:** Inhibition of Jak enzyme activity

**Kinase**	**Compound 1 IC**_**50**_**(nM)**	**Compound 2 IC**_**50**_**(nM)**
Tyk2	6 ± 1	<3 (N = 4)
Jak1	5 ± 0.4	10 ± 0.1
Jak2	7 ± 2	4 ± 1
Jak3	42 ± 1	41 ± 3

Values are mean and standard errors.

For values below the sensitivity of the assay; the number of replicates is given.

### Proteolysis reveals stabilization of enzyme in presence of inhibitor

**Figure 2:**
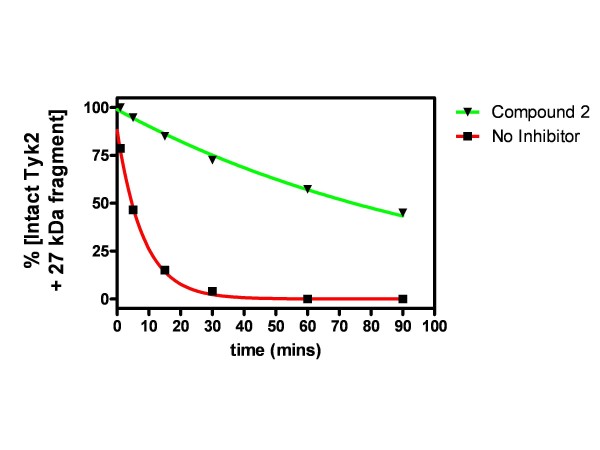
**Tyk2 is protected from proteolysis by the addition of Compound 2.** Shown are the combined, quantitated values of Tyk2 peaks of ~27 and ~29 kDa during digestion with thermolysin, in the absence or presence of 30 μM Compound 2. Intact Tyk2 protein runs as ~29 kDa. The ~27 kDa form produced by thermolysin digestion is unaffected by the presence of Compound 2.

Despite not directly forming crystal contacts, we found that inclusion of an ATP-competitive inhibitor was required for formation of mouse Tyk2 crystals. To understand the importance of ligand binding to the overall stability of the enzyme, we measured the Tyk2 kinase domain’s susceptibility to proteolysis in the presence and absence of a ligand. Compound 2 (30 μM) significantly increased resistance to partial proteolysis by thermolysin (Additional file 1: Figure S1). Minor processing of the kinase domain from ~29 kDa (intact) to ~27 kDa form by thermolysin is unaffected by addition of Compound 2, suggesting that its binding in the ATP site is insufficient to prevent cleavage of one of the extreme termini of our Tyk2 kinase domain construct. However, the rate of degradation of the enzyme to smaller forms (<27 kDa) is reduced by 13-fold (Figure 2). Like all protein kinases, the ATP binding site for Tyk2 is nestled between the N-terminal and C-terminal lobes. Our proteolysis data suggest that the conformational flexibility of the kinase, other than a ~2 kDa portion of one terminus (likely the N-terminus based on the disorder seen from residue 870–884 in the crystal structure described below), is decreased by the binding of these 3-aminoindazole inhibitors. The ability of Compound 1 to enable robust Tyk2 crystallization may be related, as inhibitor-induced decreased flexibility may favorably affect entropic loss during crystal nucleation and growth.

### Tyk2 crystal structure

**Figure 3:**
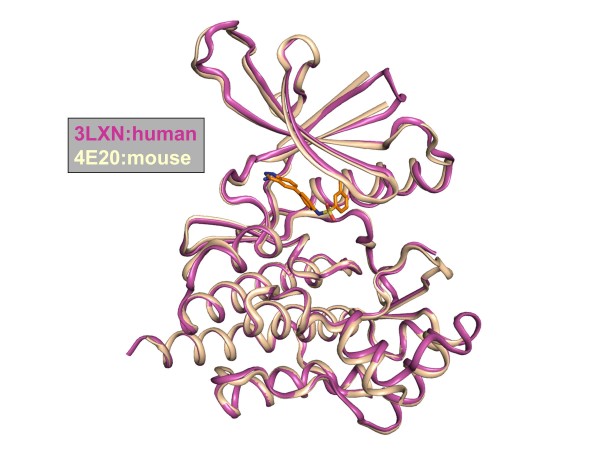
**Overlay of 4E20 (beige) with previously reported structure 3LXN (rose):** The overall structural fold is preserved when comparing the two structures with an r.m.s.d of 0.5 Å. Compound 1 is shown in orange.

**Figure 4:**
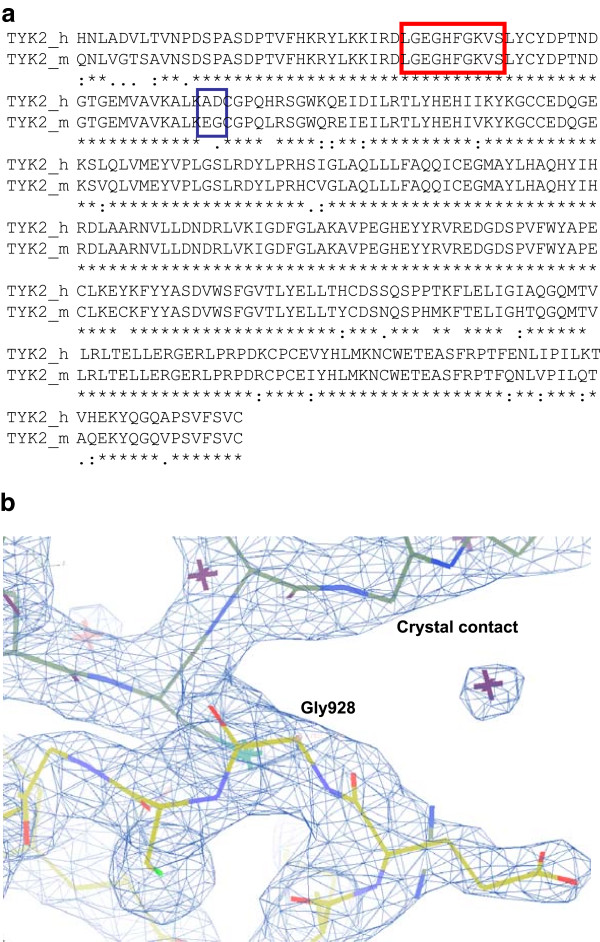
**Tyk2 sequence variation enables novel crystal contacts.a**: Sequence alignment of human and mouse Tyk2 using CLUSTALW [38]. The residues highlighted in the red box are the glycine rich loop. **b**: Location of mouse Tyk2 surface residue Gly928 (Asp935 in human Tyk2) permits a close, van der Waals crystal contact. (Picture generated with COOT) [36].

The overall structure of the mouse Tyk2 kinase domain is very similar to that of the recently reported human Tyk2 kinase domain complexed to CP-690,550 (PDB entry 3LXN; r.m.s.d 0.5 Å) (Figure 3) [27]. Two particular sequence differences between mouse and human Tyk2 may enable the crystallization of the mouse ortholog. The structure revealed that the substitution of Glu927 and Gly928 for Ala934 and Asp935 in human Tyk2 permits Gly928 to form a close, van der Waals crystal contact. Additionally, there is a potential interaction between Glu927 and Arg1132 in an adjacent molecule in the crystal lattice. Primarily due to steric clashes, a similar crystal packing would not be possible in human Tyk2. Figure 4a illustrates the sequence alignment between the mouse and human Tyk2 catalytic domains, and Figure 4b provides a view of this crystal contact.

**Figure 5:**
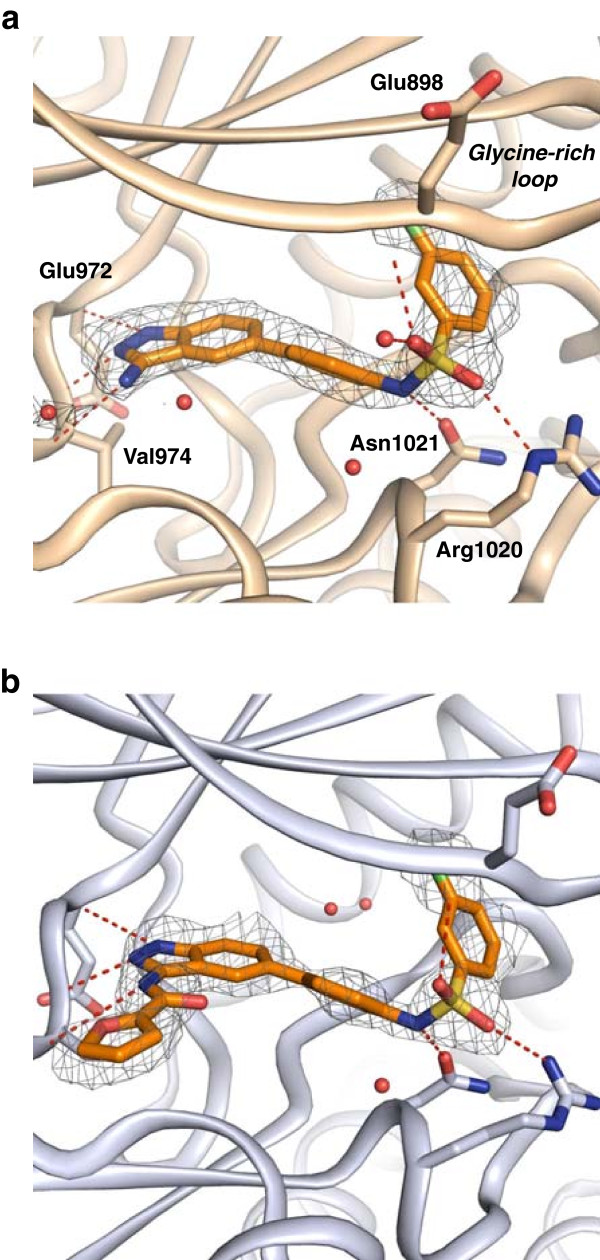
**Mouse Tyk2/inhibitor crystal structures. a**: Structure of Compound 1 (orange) complexed to mouse Tyk2 with experimental Fo–Fc electron density contoured at 2σ. The 3-aminoindazole moiety displays three hinge interactions, the *m*-chlorophenyl group is located underneath the glycine rich loop and the phenyl sulfonamide linker is stabilized by surrounding Arg1020, Asn1021 and Glu898, residues. **b**: Structure of the Tyk2/Compound 2 complex shows similar interactions with experimental Fo–Fc electron density contoured at 2σ.

The mouse Tyk2/Compound 1 co-crystal structure is illustrated in Figure 5a. The 3-aminoindazole core serves as a canonical hinge binder, forming three hydrogen bonding interactions with hinge residues Glu972 and Val974. The inhibitor’s central phenyl group linker positions the sulfonamide chlorophenyl group under the glycine rich loop. Figure 4a shows that the chlorophenyl moiety occupies a distinct hydrophobic pocket proximal to the DFG pocket. The placement of this moiety is guided by the sulfonamide linkage’s stabilizing interactions with the NH backbone of Glu898 in the glycine-rich loop, and conserved residues Asn1021 and Arg1020. The structure of Tyk2 and Compound 2 is illustrated in Figure 5b. The binding mode and trajectory of the chlorophenyl is identical to that of Compound 1 and, as a result, the glycine-rich loop adopts the same conformation in both structures. The furan substituent on the hinge-binding 3-aminoindazole core was well-ordered, providing clear evidence that the inhibitor soak was successful. The furan occupies the extended hinge region, sandwiched between Arg894 and Gly977.

**Figure 6:**
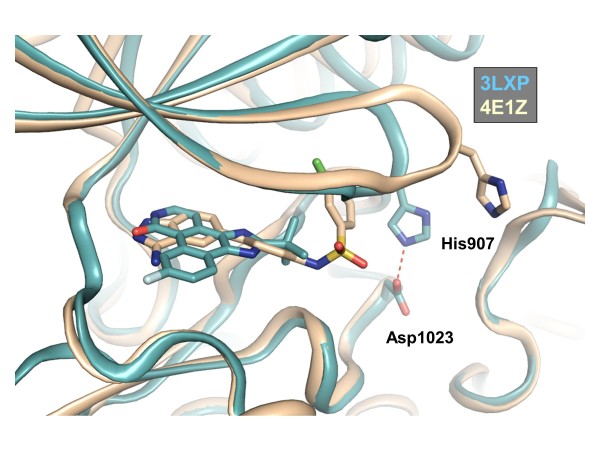
**3-Aminoindazoles stabilize an open conformation of the Tyk2 glycine-rich loop.** The chlorophenyl group of Compound 1 occupies hydrophobic pocket under the glycine rich loop. Overlay of mouse Tyk2 /Compound 2 (tan) and human Tyk2/CMP-6 (PDB entry 3LXP, cyan) structures. Deviation in the positioning of the glycine-rich loop tip is demonstrated.

One notable secondary structure difference between the co-crystallized mouse Tyk2/Compound 1 complex and the recent human Tyk2/CMP-6 complex (PDB entry 3LXP) occurs at the tip of the glycine-rich loop. An overlay shows that Compound 1 induces a ~4 Å upward shift in the loop (Figure 6), resulting in a more open active site conformation. In a recent review, it was suggested that the conformational dynamics of the glycine-rich loop may differ within the Jak family [28]. This may be due to sequence diversity in the glycine-rich loops of Jak1, Jak2, Jak3 and Tyk2. Specifically, in Tyk2 and Jak1, a “collapsed” glycine-rich loop conformation may depend upon an interaction between a histidine residue and a proximal aspartate (His907 and Asp1023 in human Tyk2). These residues are absent in Jak2 and Jak3. In the mouse Tyk2 structures, complexed to either Compound 1 or Compound 2, the steric bulk of the sulfonamide chlorophenyl moiety occupies substantial hydrophobic space under the glycine-rich loop and would potentially disrupt the His/Asp “glycine-rich loop lock,” thereby creating a larger active site pocket. While there are crystal contacts near the loop, we believe, based on multiple crystal structures determined with different soaked inhibitors (data not shown), that the loop conformation is driven mainly by the ligand. We cannot rule out, however, that some differences in loop conformation between human and mouse Tyk2 may be driven by crystal packing. Despite a more open conformation, we hypothesize that mouse Tyk2 was able to crystallize with these inhibitors because the chlorophenyl moiety stabilized the flexible glycine-rich loop. Inclusion of the chloro group also improves potency by roughly 10-fold in an enzyme activity assay (data not shown).

## Conclusion

After exploring multiple expression constructs, including trials with several orthologs and mutations, we developed a method for rapid structure determination of Tyk2/inhibitor complexes suitable for iterative SBDD. We obtained crystals with a kinase-inactive form of the mouse Tyk2 catalytic domain, only in the presence of an ATP-competitive 3-aminoindazole inhibitor. This crystal form provided a robust inhibitor soaking platform that enabled structure-based drug design of Jak inhibitors. We showed by partial proteolysis that binding of a 3-aminoindazole dramatically stabilizes Tyk2 relative to the unliganded enzyme. The resulting two crystal structures demonstrated the ability of these inhibitors to stabilize the glycine-rich loop and thus to promote conformational homogeneity. Our work indicates that compound-dependent stabilization of proteins targeted for crystallography can be a useful strategy to enable structure-based drug design.

## Methods

### Mouse Tyk2 cloning and purification

Mouse Tyk2 (UNIPROT Q9R117) was cloned and expressed in *Sf*9 insect cells. The coding region of the catalytic domain of mouse (mu) Tyk2 (residues 870–1170) was PCR sub-cloned into pDONR221 using the BP reaction of the Gateway® (Invitrogen/LTI) cloning system. The muTyk2 catalytic domain was immediately preceded by a primer encoded Tobacco Etch Virus (TEV) protease cleavage site. The resulting Tev-muTyk2 (870–1170) was modified using the Quick-Change Site-Directed Mutagenesis System (Stratagene) to replace Asp1016 with Ala. After sequence confirmation, Tev-muTyk2 (870–1170) Asp1016Ala was sub-cloned into the pDEST20 expression vector (Invitrogen/LTI) using the Gateway® LR reaction to create an in-frame fusion with an amino-terminally encoded Glutathione S-Transferase (GST). The resulting expression plasmid, pDEST20 GST-Tev-muTyk2 (870–1170) Asp1016Ala, was confirmed by DNA sequencing. The entire expression cassette was then transferred to baculovirus. Virus production and amplifications were carried out according to Invitrogen/LTI Bac-To-Bac system instructions.

High titer virus stocks were made as recommended and used to infect *Sf*9 cells, cultured in Sf900II medium (Invitrogen/LTI) at 27.5°C, at an estimated M.O.I of 2.5 to 5.0. Infected cells were harvested by centrifugation at 48 h post-infection, which was optimal for Tyk2 protein expression.

Mouse Tyk2 (870–1170) Asp1016Ala protein pellet was suspended on ice in lysis buffer containing Buffer A (50 mM HEPES pH 7.5, 500 mM NaCl, 10% glycerol, and 1 mM adenosine) in addition to 2X protease inhibitor tablets (Roche Applied Science). The resulting mixture was sonicated three times with 20 second blasts. The mixture was then added to 10 mL of GST affinity resin for 2.5 h, centrifuged at 1,000 × g, and washed. TEV protease was added to the resin and the mixture was loaded into a column; it was incubated for 2 h at room temperature, and additionally overnight at 4°C. The protein was then washed off with Buffer A and collected as monitored by A_280_. The pooled protein was concentrated and dialyzed overnight into 50 mM HEPES pH 7.5, 100 mM NaCl, 5 mM DTT, 1 mM ADP. The resulting protein was pooled (0.5 mg/mL) and used directly for crystallization trials.

### Mouse Tyk2 crystallization

Mouse Tyk2 protein (0.5 mg/mL) was incubated with Compound 1 (0.1 mM) and concentrated to 10 mg/mL. After 3–4 days, protein crystals grew using the vapor diffusion method in sitting drop plates under the following condition: 4.3–4.7 M ammonium formate, 100 mM Tris pH 8.0. Crystals were subsequently used for soaking inhibitors of interest. Compound 2 was soaked into the Tyk2 crystals by adding 1 μM inhibitor (final concentration) to a 100 μL well of harvest mother liquor. Crystals were frozen from mother liquor solution containing 20% glycerol.

### Mouse Tyk2 structure determination

X-ray diffraction data from mouse Tyk2/Compound 1 crystals were collected at the IMCA beamline 17ID at the Advanced Photon Source in Argonne, IL. The crystals were maintained at 100 K with an Oxford Cryosystems Cryostream cooler during data collection. A total of 180 frames were collected at an oscillation range of 1.0°. The data were processed with the HKL2000 suite of programs. After determining the crystal orientation, the data were integrated with DENZO, scaled/merged with SCALEPACK, placed on an absolute scale and reduced to structure factor amplitudes with TRUNCATE. Five percent of the unique reflections were assigned randomly to the “free” set, for calculation of the free R-factor (R_free_) [29]. The remaining 95% of the reflections constituted the “working” set for calculation of the R-factor (R). The x-ray diffraction data and refinement statistics are summarized in Table 2.

A maximum likelihood molecular replacement solution was determined using the program PHASER [30-32]. One Tyk2 monomer was located in the asymmetric unit, in the space group *P*3_1_21. The search model was a crystal structure of Jak2 reported previously (PDB entry 2B7A). Coordinates were generated based on the molecular replacement solution. The refinement of the Tyk2/Compound 1 complex crystal structure began with the molecular replacement solution coordinates. Rigid-body refinement was conducted by the program REFMAC [33] in the CCP4 suite of programs, which resulted in the following statistics at 2.6 Å: R 0.39 (R_free_= 0.39). Experimental Tyk2 and inhibitor electron density was observed. Manual building of Compound 1 into the density was attempted using the molecular graphics program O [34] and examination of 2Fo–Fc and Fo–Fc electron-density maps. The refinement program REFMAC was used for iterative rounds of restrained refinement [33]. Final rounds of refinement were conducted using AUTOBUSTER (Global Phasing) [35], which added water molecules to the final model, resulting in the following statistics: R 0.199 (R_free_= 0.232). Final refinement statistics are shown in Table 3. The quality of all models was evaluated using COOT [36]. The co-crystal structure of Compound 2 complexed to Tyk2 was solved by molecular replacement using the Tyk2/Compound 1 structure as a probe. An origin shift of [0 0 ½] was applied to match the Compound 1 coordinates. DETWIN [37] was used with a twinning fraction of 0.24 to improve refinement statistics. Final rounds of refinement were conducted using AUTOBUSTER (Global Phasing) [35]. Final refinement statistics are listed in Table 3.

**Table 3:** Crystallographic statistics for Tyk2/Compound 1 and Tyk2/Compound 2 complexes

**Structure**	**Tyk2/ Compound 1**	**Tyk2/ Compound 2**
PDB entry	4E20	4E1Z
Data Collection		
Resolution (Å)	50.0–2.60	20.0–2.50
(Highest shell, Å)	2.64–2.60	2.54–2.50
Space Group	P3_1_21	P3_1_21
Unit Cell Lengths (*a*, *b*, *c*; Å)	67.3, 67.3, 154.9	68.0, 68.0, 153.0
Unique reflections	13,121	14,608
Mosaicity (°)	0.247	0.642
Overall Statistics (Highest Shell)		
R_sym_ (%)	0.085 (0.511)	0.055 (0.480)
* I/σ*_*I*_	8.9 (2.5)	9.2 (2.3)
Data completeness (%)	99.9 (100)	98.3 (97.4)
Mean multiplicity	6.9 (5.1)	6.8 (4.7)
Refinement		
Reflections used in refinement	13,072	13,973
R_cryst_ (%)	19.9	19.8
R_free_ (%)	23.2	24.3
R.m.s. deviations, bond lengths (Å), bond angles (°)	0.010, 1.15	0.010, 1.20
Ramachadran plot		
Most favored (%)	96.4	96.1
Allowed (%)	2.2	3.2
Disallowed (%)	1.5	0.7

### Time-resolved fluorescence resonance energy transfer (trFRET) kinase activity assays

#### Tyk2

6 nM purified human Tyk2 enzyme (residues 880–1185; expressed in *Sf*9 cells with a cleavable *N*-terminal histidine tag and a *C*-terminal Flag tag and purified by affinity chromatography) was mixed with 2 μM peptide substrate (biotin-TYR1, sequence: Biotin-(Ahx)-GAEEEIYAAFFA-COOH) at varying concentrations of inhibitor in reaction buffer: 50 mM MOPSO pH 6.5, 10 mM MgCl_2_, 2 mM MnCl_2_, 1 μM ATP, 2.5 mM DTT, 0.01% BSA, and 0.1 mM Na_3_VO_4_. After 60 min incubation at room temperature, the reaction was quenched by addition of EDTA (final conc. 100 μM) and developed by addition of revelation reagents (final approximate concentrations: 30 mM HEPES pH 7.0, 0.06% BSA, 0.006% Tween-20, 0.24 M KF, 80 ng/mL PT66K (europium-labelled anti-phosphotyrosine antibody, cat #61T66KLB, Cisbio, Bedford, MA) and 3.12 μg/mL SAXL (Phycolink streptavidin-allophycocyanin acceptor, cat #PJ25S, Prozyme, San Leandro, CA)). The developed reaction was incubated in the dark either at 4°C overnight or at room temperature for ~1 h, then read with a time-resolved fluorescence detector (Rubystar, BMG Labtech) using a 337 nm laser for excitation and emission wavelengths of 620 nm and 665 nm. Within the linear range of the assay, this signal is directly related to phosphorylated product and was used to calculate IC_50_ values. Typically, seven-point inhibitor dilutions (5-fold; from 50 μM to 0.0032 μM) were used. IC_50_ values were calculated by fitting the following equation:

(1) $$Y=Y_{\text{max}}*IC_{50}/\left(IC_{50}+\left[I\right]\right)$$

where [I] is total inhibitor concentration, Y is the percentage of activity (relative to that seen in no-inhibitor control) at a given inhibitor concentration, and Y_max_ is the maximum activity generated in the absence of inhibitor.

#### Jak1, Jak2 and Jak3

Purified Jak2 and Jak3 were purchased from Upstate/Millipore (cat numbers #14–640 and #14–629). Jak1 (residues 845–1142) was expressed in *Sf*9 cells as a GST fusion protein and purified in-house, and used in trFRET kinase assays in the reaction buffer described above. Jak1 and Jak3 assays used substrate peptide biotin-TYR2 (Biotin-(Ahx)-AEEEYFFLFA-amide), while the Jak2 assay used biotin-TYR1 (Biotin-(Ahx)-GAEEEIYAAFFA-COOH).

### Proteolysis experiments

Mouse Tyk2 kinase domain (0.25 mg/mL, residues 870–1170; Asp1016Ala mutant) was incubated for 0 to 90 minutes with thermolysin (0.25 mg/mL) at room temperature in 50 mM HEPES pH 6.7, 150 mM NaCl, 5% glycerol, and 2.5 mM CaCl_2_ in the presence and absence of 30 μM Compound 2. EDTA (final conc 100 mM) was used as stop solution to quench the proteolysis reactions. Samples were separated by use of a Caliper LC90 system and the remaining substrate and product bands were quantitated. Intact (undigested) Tyk2 ran in this system at ~29 kDa. Addition of thermolysin yielded a partial digestion product of ~27 kDa (Additional file 1: Figure 1) within 5 minutes, which was unaffected by addition of Compound 2. Subsequent degradation products were strongly influenced by the presence of Compound 2 (Additional file 1: Figure S1). Therefore the sum of the intensities of the 29 and 27 kDa peaks was used as a measure of inhibitor-dependent resistance to thermolysin digestion. Addition of Compound 2 did not alter the measurable digestion of a BSA control by thermolysin in the same buffer (Additional file 1: Figure S2a), indicating that Compound 2 was not an inhibitor of thermolysin protease activity. Thermolysin amounts were found to be unchanged over the course of the experiment, providing a convenient loading control (Additional file 1: Figure S2b).

### Compound synthesis

#### *N*-[4-(3-Amino-1*H*-indazol-5-yl)-phenyl]-3-chloro-benzenesulfonamide (Compound 1)

(See Figures 1, 8)

Step a. 4-(3-Cyano-4-fluoro-phenyl) aniline

(Figure 7)

**Figure 7:**
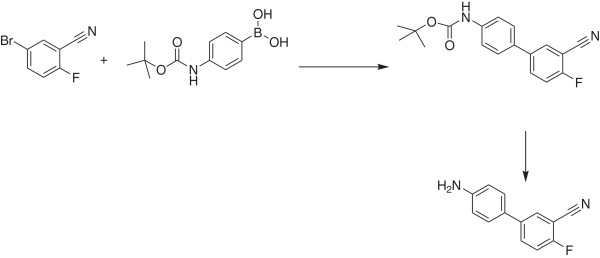
Step a. Synthetic route to 4-(3-Cyano-4-fluoro-phenyl) aniline.

A mixture of 5-bromo-2-fluorobenzonitrile (2.185 g, 10.93 mmol), 4-(*tert-*butoxycarbonylamino)phenylboronic acid (2.59 g, 10.93 mmol), *tetrakis*-(triphenylphosphine)palladium (0.758 g, 0.656 mmol) and cesium carbonate (10.68 g, 32.8 mmol) in 1,2-dimethoxyethane (40.0 ml) and water (20.00 ml) was heated at 80°C, under N_2_ for about 2.5 hours. to give a dark red solution. The reaction mixture was diluted with ethyl acetate (100 mL) and water (100 mL) then stirred for about 10 minutes. The aqueous layer was separated and re-extracted with ethyl acetate (2 × 50 mL). The combined organic phases were washed with water (3 × 50 mL) then dried over anhydrous magnesium sulfate and filtered. The solvent was removed to yield a brown solid (4.12 g) which was triturated with boiling 30–60°C petroleum ether (40 mL), cooled and collected. This solid was further washed with 30–60°C petroleum ether (2 × 20 mL) and dried to yield crude *N-tert-butoxycarbonyl-4-(3-cyano-4-fluoro-phenyl) aniline* as a pale brown solid (3.86 g). The crude protected aniline was dissolved in dichloromethane (80 mL) then treated with trifluoroacetic acid (14.0 mL, 182 mmol) and stirred at ambient temperature for about 3 hours. Water (100 mL) was added and the aqueous acidic layer was separated. The remaining organic layer was further extracted with aqueous hydrochloric acid (5 N, 4 × 60 mL). The combined acidic aqueous layers were washed with dichloromethane (3 × 50 mL), cooled with ice then basified by the addition of solid sodium hydroxide while maintaining a temperature of below 15°C. The resulting aqueous layer was extracted with ethyl acetate (4 × 75 mL) and the combined organic layers were washed with water (3 × 80 mL), dried over anhydrous magnesium sulphate, filtered and concentrated to yield a mauve solid (2.1 g). This was crystallized from ethyl acetate (8 mL) and 30–60°C petroleum ether (32 mL), filtered, washed with 30–60°C petroleum ether (2 × 15 mL) and dried to yield *4-(3-cyano-4-fluoro-phenyl) aniline* as a pale mauve powdery solid (1.84 g, 80% yield); ^1^ H NMR (DMSO-*d*_*6*_) δ 6.62 (dd, 2 H), 7.41 (dd, 2 H), 7.50 (t, 1 H), 7.93 (m, 1 H), 8.06 (dd, 1 H); LC/MS (5–60% gradient of acetonitrile in 10 mM aqueous ammonium acetate over 1.5 min, 60–95% gradient of acetonitrile in 10 mM aqueous ammonium acetate over 2.5 min with a hold at 95% acetonitrile for 1.2 min (1.3 mL/min flow rate) using a Vydac Genesis C8 column (4.6 × 30 mm, 4 μm particle) with diode array (DAD), evaporative light scattering (ELSD) and positive/negative electrospray ionization detection) R_t_ = 2.43 min; MS *m/z*: 254 (M + H + CH_3_CN)^+^.

Step b. *N*-[4-(3-Amino-1 *H*-indazol-5-yl)-phenyl]-3-chloro-benzenesulfonamide **(Compound 1)**

(See Figure 8)

**Figure 8:**
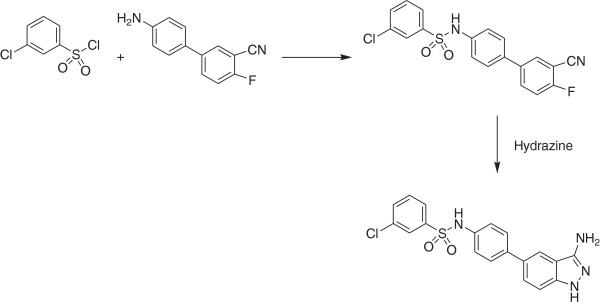
Step b. Synthetic route to Compound 1.

3-Chlorobenzenesulfonyl chloride (0.563 mL, 4.0 mmol, 2 equivalents, Aldrich) was added to a mixture of 4-(3-cyano-4-fluoro-phenyl)aniline (424 mg, 2.0 mmol) in 1,4-dioxane (10 mL) and *N,N*-diisopropylethylamine (1.04 mL). The resulting mixture was stirred at ambient temperature for about 16 hours then dispensed into a microwaveable tube and anhydrous hydrazine (2 mL) was added. The tube was sealed and heated at 140°C for 15 minutes in a microwave. The solvent was removed under reduced pressure and the residue was purified by reverse-phase preparative chromatography (using a gradient of 5 to 100% acetonitrile in 0.05 N aqueous ammonium acetate) followed by chromatography over silica gel using a mixture of 9:1 dichloromethane:methanol as the eluent to afford *N-[4-(3-amino-1 H-indazol-5-yl)-phenyl]-3-chloro-benzenesulfonamide* (113 mg, 14% yield); ^1^ H NMR (DMSO-*d*_*6*_) δ 5.39 (m, 2 H), 7.17 (m, 2 H), 7.26 (d, 1 H), 7.47 (dd, 1 H), 7.56 (m, 3 H), 7.72 (m, 2 H), 7.79 (t, 1 H), 10.41 (s, 1 H), 11.40 (m, 1 H); LC/MS (5–95% gradient of acetonitrile in 10 mM aqueous ammonium acetate over 2.9 min with a hold at 95% acetonitrile for 18 min (1.3 mL/min flow rate) using a Zorbax XDB C18 column (4.6 × 50 mm, 5 μm particle) with diode array (DAD), evaporative light scattering (ELSD) and positive/negative electrospray ionization detection) R_t_ = 2.32 min; MS *m/z*: 399, 401 (M + H)^+^.

#### Furan-2-carboxylic acid {5-[4-(3-chloro-benzenesulfonylamino)-phenyl]-1 *H*-indazol-3-yl}amide (Compound 2)

(See Figure 9)

**Figure 9:**
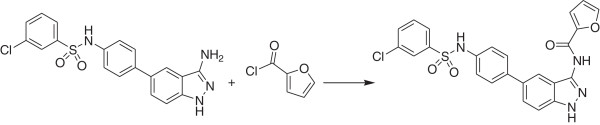
Synthetic route to Compound 2.

2-Furoyl chloride (0.123 mL, 1.254 mmol, Aldrich) was added dropwise to a solution of *N*-[4-(3-amino-1 *H*-indazol-5-yl)-phenyl]-3-chloro-benzenesulfonamide (250 mg, 0.627 mmol, Compound 1) in pyridine (6.5 mL) at about 0°C. The reaction was allowed to warm to ambient temperature and stirred for 18 hours. Ethyl acetate (20 mL) and methanol (5 mL) were added to the reaction mixture and the resulting solution was washed with water (3× 10 mL). The organic layer was dried over anhydrous magnesium sulphate and concentrated under reduced pressure. The residue was purified over silica gel eluting with a gradient of 0–100% ethyl acetate in heptane to afford *furan-2-carboxylic acid {5-[4-(3-chloro-benzenesulfonylamino)-phenyl]-1 H-indazol-3-yl}amide* (195 mg, 63% yield); ^1^ H NMR (DMSO-*d*_*6*_) δ 6.71 (s, 1 H), 7.18 (d, 2 H), 7.46 (d, 1 H), 7.54 (m, 5 H), 7.64 (m, 2 H), 7.73 (s, 1 H), 7.84 (s, 1 H), 7.91 (s, 1 H), 10.42 (s, 1 H), 10.71 (s, 1 H), 12.79 (s, 1 H); LC/MS (5–95% gradient of acetonitrile in 10 mM aqueous ammonium acetate over 2.9 min with a hold at 95% acetonitrile for 18 min (1.3 mL/min flow rate) using a Zorbax XDB C18 column (4.6 × 50 mm, 5 μm particle) with diode array (DAD), evaporative light scattering (ELSD) and positive/negative electrospray ionization detection) R_t_ = 2.47 min; MS *m/z*: 491, 493 (M-H^+^)^-^.

## Competing interests

The authors declare that they have no competing interests.

## Authors’ contributions

MAA and DWB led the Tyk2 structural biology sub-team and participated in construct design. RWD contributed to construct design. MAA and DWB solved and refined the reported crystal structures. SS made the majority of the constructs and performed some of the protein expression. DB, DM and VP purified protein, set up crystallizations, and collected diffraction data. MT and GO contributed protein characterization and purification for enzymatic assays. RS provided construct design and protein expression oversight and participated in construct design. RVT participated in construct design and supervised the structural biology and enzymology teams. ERG supervised the enzyme screening and performed the proteolysis experiments. JV, KW and NW jointly led the Tyk2 project team. NM, LW and AB conceived and synthesized the compounds. MAA and ERG jointly prepared the manuscript, in consultation with all of the co-authors. All authors have read and approved this manuscript.

## Supplementary Material

Additional file 1 **Figure S1.** Caliper LC90 “virtual gel” depiction of chromatography results with Tyk2 proteolysis using thermolysin. 0.25 mg/mL Tyk2 kinase domain was incubated with thermolysin at room temperature in 50 mM Hepes pH 6.7, 150mM NaCl, 5% Glycerol, 2.5 mM CaCl2 in the presence and absence of Compound 2. EDTA (final conc 100 mM) was used as stop solution to quench the proteolysis reactions. 8 μL of this reaction were subsequently run in the Caliper LC90 “gel chip”. Small processing of Tyk2 from ~29 kDa (intact) to ~27 kDa form by thermolysin is unaffected by addition of Compound 2, suggesting that its binding in the ATP site is insufficient to prevent processing of one of the extreme termini of our Tyk2 construct. In the absence of inhibitor, a 20 kDa fragment is generated after ~1-5 minutes and subsequently degraded. This fragment is undetectable in the similar digestion in the presence of **Compound 2**. Quantitated values of Tyk2 peaks of ~27 and ~29 kDa during digestion with thermolysin, in the absence or presence of 30 μM Compound 2 were used to monitor overall degradation rates in Figure 2. **Figure S2:** (a) Addition of **Compound 2** did not alter the measurable digestion of a BSA control (at 1 mg/mL in reaction) by thermolysin (0.5 mg/mL in reaction) in 50 mM Hepes pH 6.7, 150 mM NaCl, 5% Glycerol, 2.5 mM CaCl. This indicates that **Compound 2** was not an inhibitor of thermolysin protease activity. Quantitated values from 2 μL injected onto the Caliper LC90 are shown scaled relative to starting concentration. (b) Thermolysin levels were found to be essentially unchanged over the course of the experiment shown in Additional file 1: Figure S1 and were not affected by the addition of **Compound 2**. **Table S1:** Crystallographic constructs attempted with expression and solubility assessments. Click here for file
