# Supplementary material for: Enabling structure-based drug design of Tyk2 through co-crystallization with a stabilizing aminoindazole inhibitor
Source: BMC Struct Biol. 2012 Sep 20;12:22. doi: 10.1186/1472-6807-12-22 (PMC3478977; doi:10.1186/1472-6807-12-22)
Supplement: Additional file 1 — Figure S1. Caliper LC90 “virtual gel” depiction of chromatography results with Tyk2 proteolysis using thermolysin. 0.25 mg/mL Tyk2 kinase domain was incubated with thermolysin at room temperature in 50 mM Hepes pH 6.7, 150mM NaCl, 5% Glycerol, 2.5 mM CaCl2 in the presence and absence of Compound 2. EDTA (final conc 100 mM) was used as stop solution to quench the proteolysis reactions. 8 μL of this reaction were subsequently run in the Caliper LC90 “gel chip”. Small processing of Tyk2 from ~29 kDa (intact) to ~27 kDa form by thermolysin is unaffected by addition of Compound 2, suggesting that its binding in the ATP site is insufficient to prevent processing of one of the extreme termini of our Tyk2 construct. In the absence of inhibitor, a 20 kDa fragment is generated after ~1-5 minutes and subsequently degraded. This fragment is undetectable in the similar digestion in the presence of Compound 2. Quantitated values of Tyk2 peaks of ~27 and ~29 kDa during digestion with thermolysin, in the absence or presence of 30 μM Compound 2 were used to monitor overall degradation rates in Figure 2. Figure S2: (a) Addition of Compound 2 did not alter the measurable digestion of a BSA control (at 1 mg/mL in reaction) by thermolysin (0.5 mg/mL in reaction) in 50 mM Hepes pH 6.7, 150 mM NaCl, 5% Glycerol, 2.5 mM CaCl. This indicates that Compound 2 was not an inhibitor of thermolysin protease activity. Quantitated values from 2 μL injected onto the Caliper LC90 are shown scaled relative to starting concentration. (b) Thermolysin levels were found to be essentially unchanged over the course of the experiment shown in Additional file 1: Figure S1 and were not affected by the addition of Compound 2. Table S1: Crystallographic constructs attempted with expression and solubility assessments. [file 1472-6807-12-22-S1.doc]

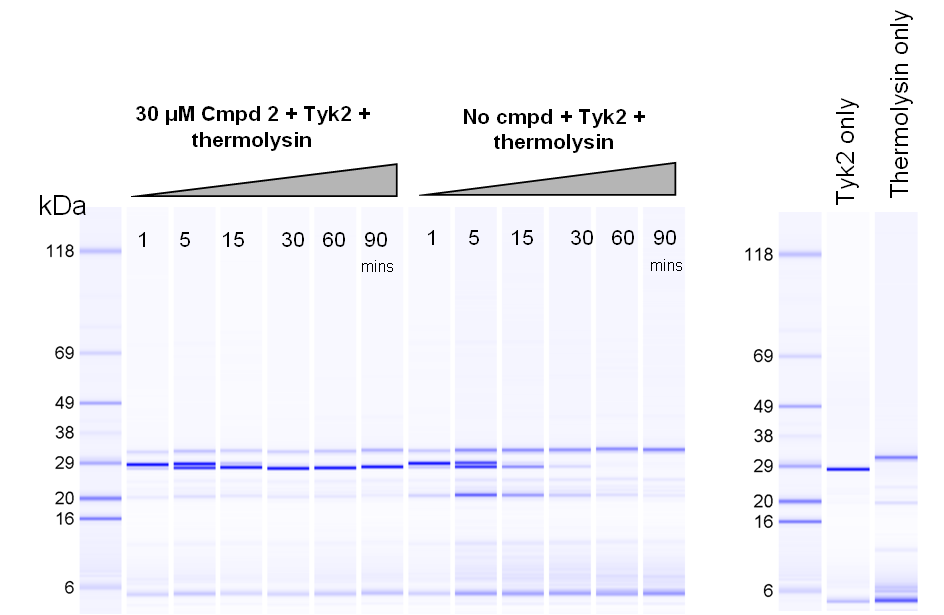


**Supplemental Figure 1:** Caliper LC90 “virtual gel” depiction of chromatography results with Tyk2 proteolysis using thermolysin.

0.25 mg/mL Tyk2 kinase domain was incubated with thermolysin at room temperature in 50 mM Hepes pH 6.7, 150mM NaCl, 5% Glycerol, 2.5 mM CaCl2 in the presence and absence of **Compound 2**. EDTA (final conc 100 mM) was used as stop solution to quench the proteolysis reactions. 8 μL of this reaction were subsequently run in the Caliper LC90 “gel chip”.

Small processing of Tyk2 from ~29 kDa (intact) to ~27 kDa form by thermolysin is unaffected by addition of Compound 2, suggesting that its binding in the ATP site is insufficient to prevent processing of one of the extreme termini of our Tyk2 construct. In the absence of inhibitor, a 20 kDa fragment is generated after ~1-5 minutes and subsequently degraded. This fragment is undetectable in the similar digestion in the presence of **Compound 2**. Quantitated values of Tyk2 peaks of ~27 and ~29 kDa during digestion with thermolysin, in the absence or presence of 30 μM Compound 2 were used to monitor overall degradation rates in Figure 2.

a

b

**Supplemental Figure 2:**

(a) Addition of **Compound 2** did not alter the measurable digestion of a BSA control (at 1 mg/mL in reaction) by thermolysin (0.5 mg/mL in reaction) in 50 mM Hepes pH 6.7, 150 mM NaCl, 5% Glycerol, 2.5 mM CaCl. This indicates that **Compound 2** was not an inhibitor of thermolysin protease activity. Quantitated values from 2 μL injected onto the Caliper LC90 are shown scaled relative to starting concentration.

(b) Thermolysin levels were found to be essentially unchanged over the course of the experiment shown in Supplemental Figure 1 and were not affected by the addition of **Compound 2**.

**Supplemental Table 1:** Crystallographic constructs attempted with expression and solubility assessments:

| **Construct** | **System** | **Expression** | **Solubility** |
| --- | --- | --- | --- |
| **HUMAN** |  |  |  |
| **His-Tev-Tyk2 1-1187** | **BV** | **High** | **Low** |
| **His-Tev-Tyk2 880-1175** | **BV** | **High** | **Low** |
| **His-Tev-Tyk2 880-1185** | **BV** | **Medium** | **High** |
| **His-Tev-Tyk2 891-1175** | **BV** | **High** | **Low** |
| **His-Tev-Tyk2 891-1185** | **BV** | **High** | **Low** |
| **GST-Thr-Tev-Tyk2 (1-1187)** | **BV** | **High** | **Low** |
| **GST-Thr-Tev-Tyk2 (880-1185)** | **BV** | **Medium** | **Medium** |
| **GST-Thr-Tev-Tyk2 (880-1185)-His** | **BV** | **Medium** | **Low** |
| **His-Tev-Tyk2 (880-1185)-Flag** | **BV** | **Medium** | **High** |
| **His-Tev-Tyk2(889-1097)-Abl(436-446) Tyk2(1123-1174)** | **BV** | **High** | **Low** |
| **His-Tev-Tyk2(889-1097)-Abl(436-446) Tyk2(1123-1179)** | **BV** | **High** | **Low** |
| **His-Tev-Tyk2(889-1097)-Abl(436-446) Tyk2(1123-1187)** | **BV** | **High** | **Low** |
| **His-Tev-Tyk2 (880-1185)-Flag** | **E. coli** |  |  |
| **GST-Thr-huTyk2 (870-1180)** | **BV** | **Medium** | **Medium** |
| **GST-Thr-huTyk2 (870-1180) D1023A** | **BV** |  |  |
| **Tyk2 (870-1180)-FLAG** | **BV** | **Medium** | **High** |
| **Tyk2 (870-1180) KD-FLAG** | **BV** | **High** | **Medium** |
| **GST-Tev-huTyk2 (589-1185)** | **BV** | **High** | **Low** |
| **GST-Tev-huTyk2 (589-1175)** | **BV** | **High** | **Low** |
| **GST-Tev-huTyk2 (450-1185)** | **BV** | **High** | **Low** |
| **GST-Tev-huTyk2 (450-1175)** | **BV** | **High** | **Low** |
| **GST-Tev-huTyk2 (589-1185) D1023A** | **BV** | **High** | **Low** |
| **GST-Tev-huTyk2 (589-1175) D1023A** | **BV** | **High** | **Low** |
| **GST-Tev-huTyk2 (450-1185) D1023A** | **BV** | **Low** | **Low** |
| **GST-Tev-huTyk2 (450-1175) D1023A** | **BV** | **Low** | **Low** |
| **His-Tev-Tyk2 (880-1185)-Flag** | **BV** |  |  |
| **His-Tev-Tyk2 (880-1185)-D1023A-Flag** | **BV** |  |  |
| **His-Tev-huTyk2(1-439)** | **E. coli** | **Medium** | **Low** |
| **His-Tev-huTyk2(21-439)** | **E. coli** | **Medium** | **Low** |
| **His-Tev-huTyk2(1-495)** | **E. coli** | **Low** | **Low** |
| **His-Tev-huTyk2(21-495)** | **E. coli** | **Medium** | **Low** |
| **His-Tev-huTyk2(1-567)** | **E. coli** | **Low** | **Low** |
| **His-Tev-huTyk2(21-567)** | **E. coli** | **Low** | **Low** |
| **GST-Thr-MuTyk2 (870-1180)** | **BV** | **Low** | **High** |
|  |  |  |  |
| **MOUSE** |  |  |  |
| **GST-Thr-mTyk2 (870-1180) D1016A** | **BV** | **Medium** | **Medium** |
| **GST-Tev-mTyk2 (870-1170)** | **BV** | **Medium** | **Medium** |
| **GST-Tev-mTyk2 (870-1170) D1016A** | **BV** | **Medium** | **Medium** |
| **GST-Tev-mTyk2 (870-1170)** | **ID** | **Low** | **Low** |
| **GST-Tev-mTyk2 (870-1170) D1016A** | **ID** | **Medium** | **Low** |
| **GST-Thr-mTyk2 (863-1170)** | **BV** |  |  |
| **GST-Thr-mTyk2 (863-1170) D1016A** | **BV** |  |  |

**Expression:**

High = visible by Coomassie stained gel

Medium = good band by WB

Low = weak band by WB

**Solubility:**

High >50%

Medium = 10-49%

Low < 10%
